# First Insight into Exploration and Cognition in Wild Caught and Domesticated Sea Bass (*Dicentrarchus labrax*) in a Maze

**DOI:** 10.1371/journal.pone.0065872

**Published:** 2013-06-21

**Authors:** David Benhaïm, Marie-Laure Bégout, Gaël Lucas, Béatrice Chatain

**Affiliations:** 1 LERMA, INTECHMER/CNAM, BP 324, Cherbourg, France; 2 Ifremer, Laboratoire Ressources Halieutiques, place Gaby Coll, BP 7, L’Houmeau, France; 3 Station Expérimentale d’Aquaculture Ifremer, Laboratoire de Recherche Piscicole de Méditerranée, Chemin de Maguelone, Palavas-Les-Flots, France; Utrecht University, Netherlands

## Abstract

European sea bass aquaculture is so recent that very little is known on the effects of the early steps of its domestication. Behavioural parameters are sensitive indicators of the domestication process since they are generally impacted as soon as the first generation. The present work compared wild-caught and domesticated sea bass juvenile swimming activity, exploration and ability to learn to discriminate between two 2-D objects associated to a simple spatial task that enabled the tested individual to visually interact with an unfamiliar congener (the reward) located behind a transparent wall at the end of one of the two arms of a maze. Ten fish from each origin were individually tested 3 times in a row during 3 days (9 trials in total). Fish were placed in a start box closed by a transparent wall located in front of two 2-D objects. Fish were filmed during 10 min after the removal of the start box wall. Different swimming variables including angular velocity, total distance travelled and velocity mean, were analyzed from videos as well as the time spent in each of 6 virtual zones including the reward zone near the congener (Cong) and the zone opposite to the reward zone (OpCong). Two learning criteria were chosen: the number of successful turns and time to reach Cong. Behavioural differences were found between domesticated and wild fish. Angular velocity was higher in wild fish while the distance travelled and the velocity mean were higher in domesticated ones. Wild and domesticated fish spent most of the time in Cong and in OpCong. No differences were seen in learning ability between wild and domesticated fish. However, our findings for learning require confirmation by further studies with larger numbers of learning sessions and experiments designed to minimise stress. This study therefore demonstrated an impact of domestication on swimming behaviour but not on spatial learning.

## Introduction

Most cultured fish have been domesticated since the beginning of the twentieth century [1] and the world aquaculture production of fish relies only on a few species that have been selected mostly upon economic and technical criteria [2]. This process is actually so recent that most cultured fishes might rather be considered as exploited captives and only a few of them would be on the threshold of becoming domesticated [3]. This statement relies on the fact that farmed fishes are little changed from their wild ancestral form and could usually be returned to the wild. However, consistent differences between wild and domesticated fish have been reviewed, the main effects being an increased growth, reproductive disturbances and alterations of behavioural traits [4], [5], [6], [7], [8], [9], [10], [11]. Among the latter, antipredator behaviour has been shown to be very sensitive to artificial rearing [4], [5], [6], [7], [8], [9], [11] and swimming or schoaling performance to be poorer in domestic stocks [12], [13]. These differences between wild and cultured fishes may be partly explained by different previous experiences [14]. Aquaculture and natural habitats are obviously very different. Farmed fishes face conditions that seem to be less challenging than natural habitats *e.g.* structurally simple environments, food easy to catch and absence of predators but they also have to adapt to high densities, restricted space, artificial and uniform food, quite frequent handling [15]. This raises the question of whether domestication could have an impact on fish cognition. Indeed, it is known that domestication influences brain size, since hatchery-reared domesticated rainbow trout (*Oncorhynchus mykiss*) have smaller brains than do wild-caught fish of the same size [16] and even first generation lab-reared guppies (*Poecilia reticulata)* can have smaller brains than wild counterparts [17].

Cognition includes perception, attention, memory formation and executive functions related to information processing such as learning and problem solving [18], [19]. The study of animal cognition has largely been centred around birds and mammals but over the last decades, it has been shown that fishes, like the rest of the vertebrates, exhibit a rich array of sophisticated behaviours and that learning plays a pivotal role in the behavioural development of fishes [18]. Several studies have shown that they have long term memories [20], [21] and that their cognitive capacity in many domains is comparable with that of non-human primates [22]. In particular, studies report that fishes use systematic exploration to extract spatial information in unfamiliar environments *e.g.* they use organized pattern of exploration when introduced into a novel environment, avoiding previously visited locations [23] and increase exploratory activity to environmental modifications [24].

Several fish species have been successfully trained to use landmark informations to solve a range of spatial tasks [21], [22]. Tasks are generally food rewarded to reinforce the learning process but shelter use, shoaling opportunity or other attributes correlated to survival can also be used [25]. Visual orientation is known to be strongly involved in the development of cognitive skills (spatial learning and problem solving) in well-structured habitats [26], [27]. More recently, it has been demonstrated that reef fish such as Ambon damselfish (*Pomacentrus amboinensis*), can discriminate between a range of visual stimuli including simple shapes drawn on a flat surface and that the choice of reward stimulus is unimportant as it can be learned [28].

European sea bass (*Dicentrarchus labrax*) is a leading species of Mediterranean aquaculture that was recently domesticated. This explains why little is known about the effects on the early steps of domestication or selection for growth apart from classical traits of commercial interest [29], [30] and personality trait differences between wild and selected fish [31]. This species has indeed demonstrated a great ability and plasticity in learning to press a lever to receive a food reward [32], [33], [34], [35], [36], [37], [38]. Under other experimental conditions *i.e.* response to acute stress, exploration and swimming activities have been compared in wild, domesticated and selected strains [31]. To the best of our knowledge, there are however no studies on sea bass focusing on cognition, and the impact of domestication on cognition.

The present work compared wild-caught and domesticated sea bass juvenile swimming activity, exploration and ability to learn to discriminate between two 2-D objects associated to a reward (visual contact with an unfamiliar congener) in a maze. The choice for an unfamiliar fish rather than a familiar one was driven by several reasons. First, two unfamiliar sea bass separated by a transparent barrier allowing only for visual contact have already been shown to spend most of their time in the zone nearest to this latter [39], [40]. Secondly, in this case, compared to chemical stimuli or multimodal combination of both visual and chemical stimuli, the visual stimulus only, seemed to increase the interest in the presence of the congener on the other side [40]. Finally, it allowed using a similar stimulus *i.e.* unfamiliar fish for both wild and domesticated fish. This reward precludes olfactory cues otherwise associated with food rewards. Further, wild juvenile sea bass are gregarious and active demersal predators with a well developed visual sense that enables them to hunt and orientate in relation to the benthic substrate, preferentially composed of rocks [41]. Finally, demersal fishes often stay within a certain home range [42] and may therefore be assumed to rely on learned spatial cues [43]. Several questions were addressed by the present study:

- Are there differences in exploration and swimming activities between wild-caught and domesticated fish in a maze?
- Are sea bass juveniles able to associate 2-D objects to a reward?
- Does domestication have an impact on cognition?

## Materials and methods

### Ethical Standards

This study was conducted under the approval of the Animal Care Committee of France under the official licence of M.L. Bégout (17-010).

### Experimental Animals and Housing Conditions

Domesticated sea bass were hatched on February 20^th^ 2009 at the Aquanord SA farm in France and transferred to the experimental station of INTECHMER on February 23^rd^ 2009 (Cherbourg), when they were grown in a recirculated system. All parameters were set according to the protocol used by Aquanord hatchery except for the temperature that was 15.2±0.5°C. The temperature usually reaches 21°C in a sea bass hatchery but here it was intentionally maintained lower to avoid creating large size differences with the wild stock that was thought to be captured later according to the natural hatching conditions.

Wild sea bass juveniles were captured off the Mediterranean coast of France (Harbour of Cap d’Agde, Southern France, 43° 58′ N; 03° 30′ 19″ E). A whole school of 560 wild fish observed from the boat was collected at low depth (280 cm). Immediately after capture they were transported to the experimental station (INTECHMER, Cherbourg) where they arrived 24 hours later, on April 15^th^. For further details, see [44].

Fish from both origins (about 400 individuals each) were later grown in open water system in two separate 2 m^3^ tanks until the beginning of this experiment which started on July 28^th^ 2010. At this date, domesticated fish were 463 days old. The age of wild-caught fish was determined from a 30-individual sample (see [44]) and was similar to that of domesticated fish. During the experimental period, light regime was 16∶8 LD (light onset at 06∶00 U.T. +1). In both tanks, temperature, salinity and oxygen level were (Mean ± SD), 18.1±0.2°C, 35.0±0.0 g L^−1^, 5.5±0.2 mg L^−1^ respectively.

Two days before the beginning of observations, all wild and domesticated fish were anesthetized with 2-phenoxyethanol (0.3 ml L^−1^) and 10 individuals from each origin were selected. Total length was (mean ± SD), 14.2±0.1 cm in domesticated fish and 14.2±0.4 cm in wild-caught ones (t-test: t = 0, p = 1); weight (mean ± SD) was 33.5±1.9 g in domesticated fish and 33.9±1.0 g in wild ones (t-test: t = −0.53, p = 0.6). Wild-caught and domesticated fish were individually placed in two 200 L tanks divided into ten numbered compartments. Additionally, a stock of ten domesticated fish of similar size were selected from another tank *i.e.* fish not familiar to the fish that were to be tested in the maze, and placed in a third 200 L tank. These fish were used as congener reward in the experiment. The three tanks were supplied with water of identical characteristics as the original tanks.

### Experimental Setup

**Figure 1:**
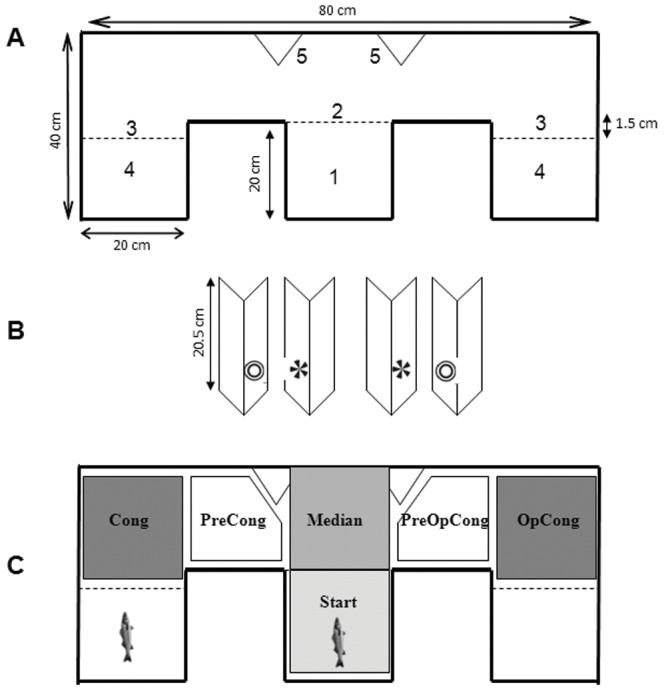
A. Schematic representation of the T-maze apparatus. Dotted lines are transparent Plexiglas walls, continous lines are white opaque plastic. The bottom of the maze is made of transparent Plexiglas. 1.Start box; 2. Removable transparent plexiglass wall; 3. Not removable transparent Plexiglas walls; 4. Compartment strictly waterproof where congeners were placed. 5. White plastic support for laminated printouts 2-D objects. B. Detail of the 4 supports and the 2-D objects. C. Virtual zones delimitation on the bottom of the maze defined for the video recordings analysis. Example of a trial where the tested fish is shown in the start box and the reward fish is shown on the left arm of the maze.

Observations were made in a dedicated room. Fish were individually tested in a maze constructed from opaque white plastic and transparent Plexiglas® (Fig. 1A). The start box was a 20×20 cm square separated from the rest of the maze by a removable transparent wall. At the end of each arm of the maze, two strictly waterproof compartments (20×18.5 cm) were also separated by a no removable transparent wall. These compartments were designed 1.5 cm shorter than the start box to ensure the tested fish was not able to see the reward before turning to the left or right side of the maze. The maze which floor was made of transparent Plexiglas® was placed on an infrared waterproof casing (1×1 m, Noldus, The Netherland) that enables video recording at low light intensity and to improve video analysis. Four white plastic supports were used to show two different laminated printouts of 2-D objects either on left or right side of the fish (Fig. 1B). Both objects (equal black and white area) were already successfully tested in previous experiment [28]. Shortly before observations, the maze was filled with water to a level maintained at 12 cm. Temperature, salinity and oxygen level were verified before and after the end of observations performed on each fish and were (mean ± SD) respectively 17.5±0.5°C, 35.0±0.0 g L^−1^, 7.8±1.5 mg L^−1^ before, 17.5±0.5°C, 35.0±0.0 g L^−1^, 7.5±1.4 mg L^−1^ after. A digital camera (Imaging Source DMK 21AUO4) with a frame rate of 30 Hz and a resolution of 640×480 pixels was positioned 42.5 cm above the water surface. Two 60 W light bulbs were horizontally placed on walls located on the left and right sides of the infrared casing. They were located 100 cm above the infrared casing and provided an indirect and homogenous lighting on the maze. The light intensity measured at the water surface of the maze was 100 Lux.

### Experimental Protocol

Before the beginning of observations, all individuals were randomly associated to one of the two 2-D objects. The position of the supports showing the 2-D objects associated with the reward was also randomly placed on left or right side of the maze arms for each individual and each trial. The reward (non-familiar fish) was then placed according to position of the 2-D object corresponding to the tested individual. The only constraint on the randomisation process was that the stimulus never appeared in the same position more than twice in a row. These objects were the most salient and detectable visual cues in the maze environment. The first tested fish was gently collected from the tank using a net and immediately placed inside a bucket closed by a cover then placed in the maze start box. After a 5 min acclimatization period, the transparent wall was removed and the video capture started. The maze was filmed during 10 min. At the end of the video recording, the individual was again placed in the start box and tested a second and a third time after a 5 min acclimatization period. In total, each individual was tested 3 consecutive times in a day, this procedure being repeated for 3 consecutive days. At the end of the 3 consecutive trials, individuals were returned to their holding tank compartments. In order to test all individuals, six days were required (two pools of 5 wild *vs.* 5 domesticated fish). The water was entirely renewed after each individual was tested and the non-familiar fish used as the reward was changed every hour to minimize stress due to confinement and handling.

### Video Analyses

The video recordings were analysed using the software EthoVision XT 5 (Noldus, The Netherlands), which allowed for six virtual zones to be defined in the maze (Figure 1C) and tracking of fish swimming behaviour.

Each video was also viewed to report the two learning criteria: first turn of the fish (left or right turn) leading to the reward zone (success) or the opposite zone (failure) (Fig. 1C) and time to reach the reward zone and/or the opposite zone.

### Behavioural Variables

Different variables of interest were chosen to analyse fish exploration and swimming:

- The time spent in each zone expressed in seconds (s): Start Box (Start), Median area (Median), Reward zone near the congener (Cong), zone opposite to the reward zone (OpCong), zone located between Median and Cong (PreCong), zone located between Median and OpCong (PreOpCong).
- The fish absolute angular velocity expressed in degree per second (Vang in ° s^−1^) was calculated by the software as followed:
  Vang_n_ = RTA_n_/t_n_ – t_n-1_ where RTA_n_ is the relative turn angle for sample n and t_n_ – t_n-1_, the time difference between the current and previous sample. Here the rate of change in direction is unsigned. The turn angle is calculated as the difference between two subsequent values for heading direction. This variable was an indicator of the amount of turns per time unit and quantified the swimming path complexity.
- The distance travelled by each fish in the maze (Dtot in mm)
- The mean velocity expressed in body lengths per second (Vel in BL s^−1^)

The last three variables quantified the fish swimming activity level in the maze.

Different variables were chosen to assess the fish learning process:

Only the very first turn was accounted to meet the successful criteria. At the end of the nine trials, the maximum score is 8 successful turns (and not 9 because the first turn at the first trial cannot be accounted as a success i.e. it was not a learned choice) if the fish eventually goes to the reward zone in the first trial, but not necessarily after its first turn choice. Similarly, if it then went to the reward zone in the second trial, the maximum possible score would be 7, and so on.

- The time to reach Cong or/and OpCong (in s)

### Statistical Analysis

All variables related to the swimming activity were compared using a repeated measures analysis of variance with Origin (wild and domesticated fish) as between-subjects factor and Trial as within-subjects factor (9 trials).

For the fish spatial distribution (time spent in each zone), since zones were not independent, a repeated measures analysis of variance, with Origin (Wild and domesticated fish) as between-subject factor and trial as a within-subject factor (9 trials) was performed for each of the two following zones: Cong and OpCong. Then a null model of space use was tested: the fish spatial distribution was compared to a theoretical homogeneous distribution in Cong and OpCong (16.6% in each zone) by a Kolmogorov–Smirnov test. Other zones (Start, Median, PreCong and PreOpCong), accordingly to their surfaces represented 66.6%.

Criteria for successful/failed turns and side-turns preference within each treatment were determined using a binomial test at a 5% level of significance.

The time to reach the reward zone was compared using a Kruskall-Wallis test taking Origin (wild vs. domesticated) and trial (9 trials) as independent variables. All statistical analyses were conducted using Statistica 8 (Statsoft, USA), and for all tests, the significant threshold was p<0.05.

## Results

### Spatial Distribution

**Figure 2:**
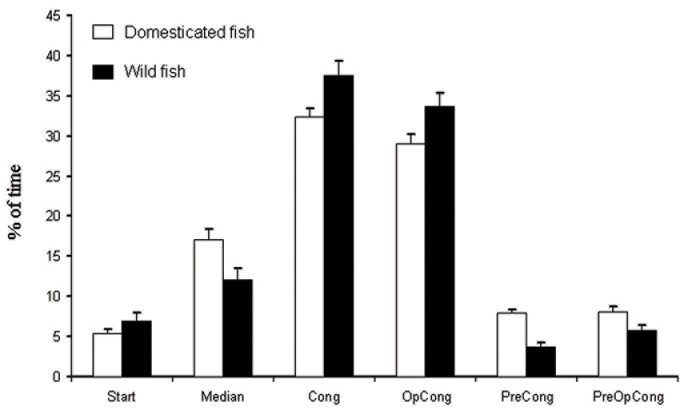
Proportion of time spent (mean ± S.E., in %) by a fish in each zone of the maze. Start : Start Box, Median: Median area, Cong: reward zone near the congener, OpCong: zone opposite to the reward zone, PreCong: zone located between Median and Cong, PreOpCong: zone located between Median and OpCong.

Wild-caught and domesticated fish spent most of the time in Cong (37 and 32% respectively) and in OpCong (33 and 29%) (Figure 2). There were no significant differences between wild-caught and domesticated fish neither in Cong nor in OpCong and any other zones. During all trials the spatial distributions of the observed fish were different from the theoretical homogeneous spatial distributions (D = 0.52, p<0.01 for Cong and OpCong).

### Swimming Activity

Vang was, although not significantly, higher in wild fish than in domesticated ones (Mean ± SE: 616.8±13.7 and 466.5±16.7° s^−1^, F_(9,10) = _2.8, p = 0.064). Differences were significant at each trial (Newman–Keuls : P<0.05 for each pairwise comparisons) except for the three first trials.

Domesticated fish travelled significantly longer distances and had higher mean velocities than wild ones (Mean ± SE: 629.9±42.3 mm and 0.07±0.00 BL s^−1^, 285.4±17.1 mm and 0.03±0.00 respectively; F_(9,10) = _3.6, p = 0.027 and F_(9,10) = _3.8, p = 0.025). Differences were significant at each trial (p<0.05 for each pairwise comparisons) except for the two first ones.

### Learning

Both wild and domesticated fish performed more successful turns than failed ones but no individual from either origin showed a significant association between the 2-D object and the reward. However three domesticated and three wild individuals showed probabilities close to the significance level (D3 and D5∶75% of successful turnings, P = 0.11; D7∶83% of successful turnings, P = 0.09; W1, W2, W5∶75% of successful turnings, P = 0.11; Table 1). When looking at individual variability (Table 1), one fish of each origin showed very low percentages of successful turns (D8∶12.5%, P = 0.03, W6∶25.0%, P = 0.11). In both cases, these fish most often performed their first turns toward the opposite stimulus (D8∶87.5%, W6∶75.0%). If these fish are considered as successful when they go toward the opposite zone, this leads to 64.6±15.1% of success in domesticated fish and 67.5±6.4% in wild ones.

**Table 1:** Learning performances in domesticated and wild individuals.

	Success (%)	P	Trials
D1	63	0.22	8
D2	63	0.22	8
D3	75	0.11	8
D4	43	0.27	7
D5	75	0.11	8
D6	50	0.27	8
D7	83	0.09	6
D8	13	0.03*	8
D9	50	0.27	8
D10	57	0.27	7
W1	75	0.11	8
W2	75	0.11	8
W3	63	0.22	8
W4	38	0.22	8
W5	75	0.11	8
W6	25	0.11	8
W7	63	0.22	8
W8	63	0.22	8
W9	63	0.22	8
W10	63	0.22	8

Successful turns are assessed by a binomial test at the 5% level of significance. Significant successful turns are signified with a single asterisk beside the probability value (P). Trials: number of accounted trials performed by each individual for the calculation of the learning performance, the maximum being 8. D: domesticated individuals; W: Wild individuals.

The time to reach the reward was (Mean ± SE) 56.8±11.1 s in domesticated fish and 47.0±10.5 s in wild ones but the difference was not significant (H_(1, 133) = _0.00, p = 0.99). There was no latency time differences between trials (H_(8, 133) = _7.9, p = 0.45).

## Discussion

The aim of this study was to assess the effects of domestication on swimming behaviour, exploration and learning abilities of juvenile sea bass in a maze. This was approached by comparing wild-caught and domesticated fish. The results showed consistent behavioural differences between fish origins. It also provided a first insight into the learning abilities for this species, such as discrimination and interpretation of abstract 2-D objects. No differences were reported in learning abilities between wild and domesticated fish. However, inter-individual differences existed that can reduce the power of statistical tests especially when using small samples in cognition-based experiments.

### Spatial Distribution and Exploratory Swimming Activity

Behavioural differences were found between domesticated and wild fish. Angular velocity was higher in wild fish while the distance travelled and the mean velocity were higher in domesticated ones. These differences between wild and domesticated fish demonstrate an impact of domestication on swimming behaviour. Difference in velocity mean and angular velocity reflected lower swimming complexity in domesticated fish that could be linked to a decrease in the vigilance threshold [45] induced by the environment experienced by cultured fish that is strikingly different from that experienced by their wild counterparts *e.g*. the physical environment is much simpler, space is restricted and migration is not possible, it is less challenging in that good quality food is readily available and fishes are protected against predators [46], [47]. On the contrary wild fish behaviour could indicate higher vigilance and then insecurity in a novel environment such as a maze. Indeed, previous studies have already shown a high frequency of turns and a slower travelling speed in solitary fish placed under similar conditions [48]. These results are also in accordance with previous studies on sea bass flight response at an early stage [44] or later stage [31].

In general, fish from both origins spent most of the time in the zone closest to the congener, the stay duration being higher in wild fish. However, differences sometimes occurred between trials. In these cases, wild and domesticated fish spent similar time in Cong and OpCong. This work was actually based on the hypothesis that fish would consider Cong as a reward zone so it is not surprising to see that fish spent generally most of the time in that zone. It confirms that social or gregarious species may greatly benefit search from social interaction. Group behaviour has already been shown to increase growth as a result of social facilitation [49], [50] and to reduce predation risk [51]. The vigilance decreases when neighbour distance decreases because information about whether other group members have detected a predator is easier to obtain from nearer individuals [52]. More recently, it has also been shown that social interaction plays an important and beneficial role in regulating the stress response in cohesive social species such as sturgeon (*Acipenser fulvescens*) [53]. The fact that wild fish spent more time in the congener zone could reinforce the hypothesis of higher vigilance in wild fish described above leading them to reduce distance from a congener. Fish from both origins also spent time in OpCong. It can be explained by the proportion of fish that failed to find the reward zone. More likely, some fish could voluntarily have avoided Cong and preferred to spend time in the opposite zone. In an experiment with two unfamiliar sea bass individuals separated by a transparent barrier, it was hypothesized that contact with the barrier could be considered as an agonistic attempt (intimidation) of the initiator towards the congener on the other side [40]. The fish that spent most of the time in the opposite zone could then be subordinates. It is well known that staying alone could be a better strategy for subordinates [54] allowing them to have a lower probability of suffering injury in an escalated contest [55]. Congener avoidance could also refer to a particular coping style because fish differed within origin in the nature of their response to the challenge [56].

### Learning Abilities

Most individuals from both origins went preferentially toward the congener zone indicating that they were able to discriminate between two 2-D objects. However, the power of the binomial test was clearly weakened by the low number of trials. Experiments on learning process usually require numerous training sessions [28], [57], [58]. This enables to compare for example the learning performances between the first and the last session. In our study, preliminary observations showed that individuals could not be tested more than 3 trials in a row. Indeed, most individuals remained immobile in the start box after 3 trials indicating high level of stress. However, the required task was quite simple compared to previously cited studies and tested fish were placed in an environment where visuals cues were mostly restricted to the 2-D objects. Indeed, the time to reach the reward was lower than 1 min. Some studies have already shown that associative learning occurs after just one simultaneous presentation of the cue and the stimulus [59] and the response can be retained for up to 2 months [60]. Here the association between the visual cue and the reward could have been learned very fast. Even though it needs to be confirmed by further research *e.g.* increasing the number of day sessions, we assume that fish from both origins would be able to discriminate between two 2-D objects with equal areas of dark and light, hereby removing therefore any remaining spurious differences in luminance between the stimuli [28] to achieve a simple task. The best learning criteria was the first turn performed by the fish. Similarly to a previous study [57] latency did not appear to be a pertinent indicator in our study because of inter-individual differences. In our study, successful fish obviously seem to use place strategy rather than response strategy because they turned according to the symbol position. The place strategy refers to animals that can learn an association between a given place and a reward [61], [62]. Previous studies have shown that fish employ multiple spatial strategies that closely parallel those described in mammals and birds [63] but one of the two strategies (place or response strategy) can be favoured by the conditions of the experiment [64].

This study also demonstrated inter-individual differences, with some fish showing a preference for the side opposite to the reward. As stated in previous section, these fish were more likely to prefer the opposite side to the reward than being “bad learners”.

### Impact of Cognition on Learning Abilities

The main focus of this work was to investigate whether learning abilities differences existed between wild and domesticated fish. Fish from both origins actually showed very similar responses to the test they were subjected to. In particular, the same proportion of “good and bad learners” was recorded. This indicates that domestication would not have a major effect on spatial orientation such as place learning in sea bass. Fish reared in tanks and cages are kept in an environment very different from the natural habitats but in both cases, they benefit from spatial learning. Wild fish need to relocate various biologically important locations such as shelter or profitable food patch [65]. To achieve this, the fish needs to monitor its location with reference to external reference points as it moves through its environment [65]. This is the case for sea bass juveniles that need to orientate in relation to visuals cues (benthic substrate composed of rocks) when becoming dermersal [41]. At the same time, cultured fish must also cognitively process the sensory information presented by the farming systems such as food location when automatically distributed [15].

Nevertheless, the captive environment was also likely to play a role in wild fish behaviour. These fish were actually kept about 400 days in captivity before the beginning of the experiment and it is well known that behaviour has both inherent and learned components shaped by rearing conditions [66]. This could explain the behavioural similarities between wild and domesticated fish that were kept in same rearing conditions.

### Conclusion

This study demonstrated behavioural differences between wild and domesticated fish facing a new environment but no difference in spatial learning. Further research is however needed on this species to confirm these results. It would be useful to increase the number of learning sessions and/or individuals in experiments designed to minimize the stress and allowing specification of the nature of the spatial learning *i.e*. testing response and place learning. Overall, this study provides the first insight into the impact of domestication on sea bass learning abilities. The findings have a potential interest for future cognition-based experiments on this species.
